# Cellulitis in adult patients: A large, multicenter, observational, prospective study of 606 episodes and analysis of the factors related to the response to treatment

**DOI:** 10.1371/journal.pone.0204036

**Published:** 2018-09-27

**Authors:** Julio Collazos, Belén de la Fuente, Alicia García, Helena Gómez, C. Menéndez, Héctor Enríquez, Paula Sánchez, María Alonso, Ian López-Cruz, Manuel Martín-Regidor, Ana Martínez-Alonso, José Guerra, Arturo Artero, Marino Blanes, Javier de la Fuente, Víctor Asensi

**Affiliations:** 1 Infectious Diseases Unit, Hospital de Galdácano, Vizcaya, Spain; 2 Infectious Diseases Unit, Hospital de Cabueñes, Gijón, Spain; 3 Infectious Diseases Unit, Hospital Universitario Central de Asturias, Oviedo, Spain; 4 Internal Medicine Service, Hospital de Povisa, Vigo, Spain; 5 Internal Medicine Service, Hospital Dr Peset, Valencia, Spain; 6 Internal Medicine Service, Complejo Hospitalario de León, León, Spain; 7 Infectious Diseases Unit, Hospital La Fe, Valencia, Spain; University of Calgary, CANADA

## Abstract

**Background:**

Cellulitis is a frequent cause of hospital admission of adult patients. Increasing prevalence of multiresistant microorganisms, comorbidities, predisposing factors and medical and surgical therapies might affect cellulitis response and recurrence rate.

**Methods:**

Prospective and observational study of 606 adult patients with cellulitis admitted to several Spanish hospitals. Comorbidities, microbiological, clinical, diagnostic, treatment (surgical and antibiotic) data were analyzed according to the cellulitis response. Good response implied cure. Poor response implied failure to cure or initial cure but relapse within 30 days of hospital discharge.

**Results:**

Mean age was 63.3 years and 51.8% were men. Poor responses were significantly associated with age, previous episodes of cellulitis, prior wounds and skin lesions, venous insufficiency, lymphedema, immunosuppression and lower limbs involvement. No differences in ESR or CRP blood levels, leukocyte counts, pus or blood cultures positivity or microbiological or imaging aspects were observed in those with good or poor responses. Regarding antimicrobials, no differences in previous exposition before hospital admission, treatment with single or more than one antibiotic, antibiotic switch, days on antimicrobials or surgical treatment were observed regarding good or poor cellulitis response. Prior episodes of cellulitis (P = 0.0001), venous insufficiency (P = 0.004), immunosuppression (P = 0.03), and development of sepsis (P = 0.05) were associated with poor treatment responses, and non-surgical trauma (P = 0.015) with good responses, in the multivariate analysis.

**Conclusions:**

Prior episodes of cellulitis, non-surgical trauma, venous insufficiency, sepsis and immunosuppression were independently associated with treatment response to cellulitis, but not the causative microorganism, the number of antimicrobials administered or its duration.

## Introduction

Cellulitis [a common type of skin and soft tissue infection] is a frequent bacterial infection of the skin and subcutaneous tissues, whose incidence is rising, and that results in substantial economic and healthcare burdens [1–7]. In fact, although mild cellulitis can be managed in the ambulatory setting by family doctors, more serious cases represent a common and progressively increasing cause of hospital admissions in developed countries, particularly among the elderly and individuals with predisposing factors or comorbidities [1–4].

In the hospital setting, patients are usually treated with intravenous (IV) antibiotics for 5–7 days, although IV or oral therapy may be prolonged up to 14 days or longer in immunosuppressed or in complex cases, depending on the response [4–6, 8]. The causative microorganism is not identified in most cases of cellulitis, but *Streptococcus pyogenes*, other streptococci and *Staphylococcus aureus* account for about three-fourths of those cases in which an agent is recovered, although the relative proportion may differ depending on the type cellulitis and the individual characteristics of the patients [3–7, 9, 10]. However, cellulitis management may be complicated if it is caused by certain agents, such as methicillin-resistant *S*. *aureus* (MRSA), a difficult-to-treat and potentially deadly microorganism requiring specific antimicrobials for its cure [6, 7, 9, 11]. Furthermore, healthcare costs are increased because of frequent hospital readmissions due to the high recurrence rate of cellulitis, which is favored by diverse local and systemic factors [3–5].

All these clinical and healthcare circumstances emphasize the value of identifying the factors leading to cellulitis for prevention purposes. In this regard, a number of predisposing factors have been described, involving mainly skin integrity, immunity or vasculature [3–5, 12–16]. Likewise, the identification of the factors related to the response to therapy would be highly desirable, in order to improve the management and outcome of these infections.

Despite its frequency, few large series of cellulitis have been published and few studies, mostly with relatively reduced sample sizes, have analyzed the interactions of the multiple potential causes leading to cellulitis, the clinical, microbiological and therapeutic aspects and the outcome. In addition, the published studies may be difficult to compare because of the variety in designs, objectives, settings, criteria, endpoints, nature and management of the infection, and type of data recorded. Finally, the vast majority of the studies are retrospective, with information obtained from medical charts or computerized databases, and their analyses have been restricted to the relatively few data gathered, obviating the confounding effect of other covariates not recorded.

Therefore large, comprehensive, prospective studies evaluating the multiplicity of factors involved in the diverse aspects of cellulitis are necessary. To this end, we have carried out a large, prospective and observational study of adult patients with cellulitis admitted to the Internal Medicine wards of several Spanish hospitals. A number of parameters, including demography, topography, predisposing factors, comorbidities, microbiological, clinical, laboratory, imaging, outcome and treatment data were recorded and analyzed, in order to identify the factors associated with poor responses to therapy.

## Patients and methods

Patients older than 18 years with cellulitis admitted to the Internal Medicine wards of Hospital Universitario Central de Asturias (HUCA), Oviedo, Hospital de Cabueñes, Gijón, Hospital Dr Peset, Valencia, Hospital de Povisa, Vigo, Complejo Hospitalario de León, and Hospital La Fe, Valencia, all in Spain, between January 1^st^ 2016 and June 30^th^ 2017 were included in the study. The diagnosis of cellulitis was primarily based on history and physical examination [3–6, 8], regardless of the use of other microbiological or imaging diagnostic procedures, and the patients were followed-up for one month after hospital discharge.The response to treatment was evaluated by the physician in charge. Good cellulitis response implied cure with absence of relapses or hospital re-admissions. Poor cellulitis response implied failure to cure, or initial response but relapse within 30 days of hospital discharge. Patients were considered as septic if fulfilled the diagnostic criteria of the Third International Consensus Definitions for Sepsis and Septic shock [17].

Many demographic, epidemiological, topographic, microbiological, clinical, laboratory, imaging, prognostic, hospitalization, and therapeutic (both surgical and antibiotic) data were collected and analyzed according to the cellulitis response to therapy. Regarding the diverse microbiological features, only positive cultures obtained from blood or drained pus collections were considered. Positive swab cultures from skin ulcers or exudates were disregarded because the risk of obtaining spurious microbiological results due to contamination.

This was an observational study, using anonymized data, in which the patients underwent routine clinical care for cellulitis, without any change in its management or specific determinations or procedures. Therefore, no formal written informed consent was obtained from the patients. The Research Ethics Committee of the Principality of Asturias granted a formal waiver of ethical approval for this study.

### Statistical analysis

Continuous variables are presented as mean, 95% CI, and categorical variables as percentage. As the distribution of continuous variables was non-Gaussian, original values underwent natural logarithmic transformation for analysis. The reported values are the result of back-transformation into the original units. Proportions were compared with the chi-square test and Fisher’s exact test, as appropriate, *t*-test was used for the comparison of continuous variables and McNemar’s test for evaluating the antibiotic changes of individual patients. A stepwise logistic regression analysis was carried out to identify the factors independently predictive of cellulitis outcome. SPSS v. 22 software (IBM Corp., Armonk, NY, USA) was used for statistical calculations. A P value <0.5 for a two-tailed test was considered statistically significant.

## Results

### All patients

A total of 606patients with cellulitis admitted to the participating hospitals from January 2016 to June 2017 were included. Table 1 shows the demographic features, as well as the maincomorbidities and potential predisposing factors for the development of cellulitis.

**Table 1:** Demography and predisposing factors of patients with cellulitis.

		All (n = 606)	Good response (n = 520)	Poor response (n = 86)	P value
**Demography & anthropometry**					
Gender	Male	314 (51.8%)	275 (52.9%)	39 (45.3%)	0.19
	Female	**292** (48.2%)	245 (47.1%)	47 (54.7%)	
Age (years)		63.43 (61.85–65.05)	62.70 (60.98–64.47)	68.03 (64.25–72.03)	0.03
Body mass index (kg/m^2^)	(n = 350)	30.00 (29.25-30-77)	29.90 (29.14–30.67)	31.07 (27.73–34.81)	0.5
Predisposing factors / comorbidities*					
Prior cellulitis	Yes	156 (25.7%)	115 (22.1%)	41 (47.7%)	<0.0001
	No	450 (74.3%)	405 (77.9%)	45 (52.3%)	
Episodes of prior cellulitis	(Only if prior cellulitis)	1.72 (1.56–1.90)	1.58 (1.42–1.76)	2.19 (1.78–2.71)	0.003
Episodes of prior cellulitis	0	450 (74.3%)	405 (77.9%)	45 (52.3%)	<0.0001
	1	79 (13.0%)	64 (12.3%)	15 (17.4%)	
	2	27 (4.5%)	23 (4.4%)	4 (4.7%)	
	3	26 (4.3%)	14 (2.7%)	12 (14.0%)	
	4 or more	24 (4.0%)	14 (2.7%)	10 (11.6%)	
Location of prior cellulitis	Same location	143 (91.7%)	104 (90.4%)	39 (95.1%)	0.5
	Other locations	13 (8.3%)	11 (9.6%)	2 (4.9%)	
Prior wounds	Yes	332 (54.8%)	289 (55.6%)	43 (50.0%)	0.3
	No	274 (45.2%)	231 (44.4%)	43 (50.0%)	
Type of wound	None	274 (45.2%)	231 (44.4%)	43 (50.0%)	<0.0001
	Skin ulcer	110 (18.2%)	80 (15.4%)	30 (34.9%)	
	Non-surgical trauma	108 (17.8%)	104 (20.0%)	4 (4.7%)	
	Surgical	34 (5.6%)	26 (5.0%)	8 (9.3%)	
	Animal bite	12 (2.0%)	11 (2.1%)	1 (1.2%)	
	Injection	11 (1.8%)	11 (2.1%)	0 (0.0%)	
	Arthropod bite	11 (1.8%)	11 (2.1%)	0 (0.0%)	
	Others	46 (7.6%)	46 (8.8%)	0 (0.0%)	
Prior skin lesions	Yes	182 (30.0%)	146 (28.1%)	36 (41.9%)	0.01
	No	424 (70.0%)	374 (71.9%)	50 (58.1%)	
Diabetes	Yes	153 (25.2%)	126 (24.3%)	27 (31.4%)	0.16
	No	453 (74.8%)	394 (75.8%)	59 (68.6%)	
Venous insufficiency	Yes	124 (20.5%)	94 (18.1%)	30 (34.9%)	0.0003
	No	482 (79.5%)	426 (81.9%)	56 (65.1%)	
Prior deep venous thrombosis	Yes	23 (3.8%)	17 (3.3%)	6 (7.0%)	0.12
	No	583 (95.9%)	503 (96.7%)	80 (93.0%)	
Edema / lymphedema	Yes	168 (27.7%)	127 (24.4%)	41 (47.7%)	<0.0001
	No	438 (72.3%)	393 (75.6%)	45 (52.3%)	
Heart failure	Yes	101 (16.7%)	82 (15.8%)	19 (22.1%)	0.15
	No	505 (83.3%)	438 (84.2%)	67 (77.9%)	
Obesity	Yes	229 (37.8%)	193 (37.1%)	36 (41.9%)	0.4
	No	377 (62.2%)	327 (62.9%)	50 (58.1%)	
Immunosuppression	Yes	70 (11.6%)	52 (10.0%)	18 (20.9%)	0.003
	No	536 (88.4%)	468 (90.0%)	68 (79.1%)	
Intravenous drug use	Yes	7 (1.2%)	6 (1.2%)	1 (1.2%)	1
	No	599 (98.8%)	514 (98.8%)	85 (98.8%)	
HIV *infection	Yes	10 (1.7%)	8 (1.5%)	2 (2.3%)	0.6
	No	596 (98.3%)	512 (98.5%)	84 (97.7%)	
Other comorbidities	Yes	452 (74.6%)	377 (72.5%)	75 (87.2%)	0.004
	No	154 (25.4%)	143 (27.5%)	11 (12.8%)	

Values are expressed as mean (95% CI) or % as appropriate.

*HIV denotes human immunodeficiency virus

The mean age was 63.3 years, and the gender distribution was balanced (men 51.8%). A total of 332 patients (54.8%) had prior wounds that could have favored the development of cellulitis, the most common of which were skin ulcers and non-surgical trauma (18.2% and 17.8%, respectively, of the patients as a whole).

About one-quarter of the patients had experienced prior episodes of cellulitis at the time of admission, mostly in the same location as the current episode (91.7%). Among other factors that could have influenced the development and/or course of the infection, diabetes was present in 25.2% of the patients, venous insufficiency in 20.5%, edema or lymphedema in 27.7%, obesity in 37.8%, immunosuppression in 11.6% and diverse other comorbidities in 74.6%.

The clinical, topographical, laboratory, imaging, hospitalization and outcome parameters are described in Table 2.

**Table 2:** Clinical, laboratory, imaging and hospitalization parameters of patients with cellulitis.

		All (n = 606)	Good response (n = 520)	Poor response (n = 86)	P value
**Clinical and topographical aspects**					
Days of symptoms		4.1 (3.8–4.5)	4.0 (3.7–4.4)	4.7 (3.8–5.8)	0.2
Temperature (°C)		37.0 (36.9–37.1)	37.0 (36.9–37.1)	37.2 (36.9–37.4)	0.1
Location of cellulitis	Lower extremities	453 (74.8%)	376 (72.3%)	77 (89.5%)	0.006
	Upper extremities	82 (13.5%)	76 (14.6%)	6 (7.0%)	
	Thorax/abdomen	26 (4.3%)	24 (4.6%)	2 (2.3%)	
	Head/neck	45 (7.4%)	44 (8.5%)	1 (1.2%)	
Exclusive or preferential side	Right	264 (43.6%)	221 (42.5%)	43 (50.0%)	0.4
	Left	292 (48.2%)	256 (49.2%)	36 (41.9%)	
	Similar	50 (8.3%)	43 (8.3%)	7 (8.1%)	
Maximum length of cellulitis (cm)	(n = 398)	20.1 (18.8–21.5)	19.9 (18.5–21.4)	21.3 (17.6–25.7)	0.5
Crepitation	Yes	9 (1.5%)	7 (1.3%)	2 (2.3%)	0.6
	No	597 (98.5%)	513 (98.7%)	84 (97.7%)	
Sepsis	Yes	65 (10.7%)	51 (9.8%)	14 (16.3%)	0.07
	No	541 (89.3%)	469 (90.2%)	72 (83.7%)	
Presence of purulent collection	Yes	164 (27.1%)	142 (27.3%)	22 (25.6%)	0.7
	No / not detected	442 (72.9%)	378 (72.7%)	64 (74.4%)	
Detection of the collection	By physical exam	104 (63.4%)	92 (64.8%)	12 (54.5%)	0.4
	Only by imaging	60 (36.6%)	50 (35.2%)	10 (45.5%)	
**Laboratory parameters**					
Blood glucose (mg/dL)		124.4 (120.9–128.0)	124.3 (120.5–128.2)	125.1 (115.4–135.5)	0.9
Blood creatinine (mg/dL)		1.03 (1.00–1.07)	1.02 (0.98–1.06)	1.11 (1.00–1.23)	0.09
Leukocyte count **(**cells x10^9^/L)		10.8 (10.4–11.2)	10.8 (10.4–11.3)	10.6 (9.4–11.9)	0.7
Neutrophil count (% of leukocytes)		75.1 (74.0–76.3)	75.3 (74.0–76.5)	74.5 (71.1–78.0)	0.6
ESR (mm/h)	(n = 161)	53.0 (47.6–59.0)	53.7 (48.0–60.1)	47.6 (32.8–69.2)	0.5
CRP (mg/L)	(n = 581)	23.6 (20.5–27.2)	23.4 (20.1–27.3)	24.7 (17.1–35.7)	0.8
**Imaging procedures**					
Imaging	Yes	277 (45.7%)	239 (46.0%)	38 (44.2%)	0.8
	No	329 (54.3%)	281 (54.0%)	48 (55.8%)	
Imaging ^a^	Only echography	147 (53.1%)	125 (52.3%)	22 (57.9%)	0.9
	Only CT	50 (18.1%)	44 (18.4%)	6 (15.9%)	
	Only MRI	18 (6.5%)	15 (6.3%)	3 (7.9%)	
	Others/combined	62 (22.4%)	55 (23.0%)	7 (18.4%)	
Imaging (single or combined) ^a^	Echography	180 (65.0%)	152 (63.6%)	28 (73.7%)	0.2
	Other than echo	97 (35.0%)	87 (36.4%)	10 (26.3%)	
				
	CT	77 (27.8%)	67 (28.0%)	10 (26.3%)	0.8
	Other than CT	200 (72.2%)	172 (72.0%)	28 (73.7%)	
				
	MRI	37 (13.3%)	31 (13.0%)	6 (15.8%)	0.6
	Other than MRI	240 (86.6%)	208 (87.0%)	32 (84.2%)	
**Hospitalization parameters & outcome**					
Days of hospital stay		7.0 (6.6–7.4)	6.9 (6.5–7.3)	8.1 (6.9–9.5)	0.052
Follow-up after discharge	Primary care	377 (63.7%)	334 (64.2%)	43 (59.7%)	0.7
	Outpatient clinic	202 (34.1%)	175 (33.7%)	27 (37.5%)	
	Others	13 (2.2%)	11 (2.1%)	2 (2.8%)	
Vital outcome	Death	18 (3.0%)	2 (0.4%)	16 (18.6%)	<0.0001
	Survival	588 (97.0%)	518 (99.6%)	70 (81.4%)	
Death related to cellulitis	Yes	5 (27.8%)	0 (0.0%)	5 (31.3%)	0.8
	No	13 (72.2%)	2 (100%)	11 (68.8%)	
Days from admission to death		7.2 (4.1–12.45)	15.0 (6.4–35.0)	6.5 (3.5–12.0)	0.3

Values are expressed as mean (95% CI) or % as appropriate

^a^Respect to patients who underwent imaging procedures

ESR denotes erythrocyte sedimentation rate, CRP C-reactive protein, CT computed tomography and MRI magnetic resonance imaging

The mean duration of symptoms at the time of admission was 4.11 days and the mean temperature 37.0°C. The most common sites of involvement were the lower (74.8%) and upper (13.5%) extremities, and the mean maximum length of the cellulitis plaque was 20.1 cm. Cellulitis evolved to sepsis in 10.7% of the patients.

From a laboratory perspective the mean leukocyte count was 10.8 cells x10^9^/L, and the mean neutrophil count 75.1%. Erythrocyte sedimentation rate (ESR) and C-reactive protein (CRP) values were usually elevated (mean 53 mm/h and 23.6 mg/dL, respectively).

Imaging procedures were used in 45.7% of the patients. The most common imaging method was echography, sole or in combination with others (65% of the patients who underwent imaging procedures), followed by CT scan (27.8%). Although physical exam was able to detect most purulent collections (63.4%), imaging methods revealed collections in the remaining 36.6% that otherwise would have been undetected.

The mean hospital stay was 7 days and almost two-thirds of the patients were sent to primary care for follow-up after discharge. Most patients (520, 85.8%) experienced a good cellulitis outcome, whereas the remainder had suboptimal or poor responses. Regarding the vital outcome, 18 patients (3.0%) died, a mean of 7.18 days from admission, and in 5 of them (27.8%) the death was related to cellulitis.

Table 3 shows the microbiological aspects.

**Table 3:** Microbiological aspects of cellulitis.

		All (n = 606)	Good response (n = 520)	Poor response (n = 86)	P value
**Culture of the purulent collection**					
Pus culture available	Yes	150 (24.8%)	128 (24.6%)	22 (25.6%)	0.8
	No	456 (75.2%)	392 (75.4%)	64 (74.4%)	
Results of culture	Positive	118 (78.7%)	101 (78.9%)	17 (77.3%)	0.9
	Negative	32 (21.3%)	27 (21.1%)	5 (22.7%)	
Positive culture	Monomicrobial	92 (78.0%)	80 (79.2%)	12 (70.6%)	0.4
	Polymicrobial	26 (22.0%)	21 (20.8%)	5 (29.4%)	
Aerobes (monomicrobial) ^a^	None	32 (21.3%)	27 (21.1%)	5 (22.7%)	0.9
	*S*. *aureus* only	45 (30.0%)	40 (31.3%)	5 (22.7%)	
	Streptococci only	18 (12.0%)	15 (11.7%)	3 (13.6%)	
	Gram-neg bacilli only	21 (14.0%)	17 (13.3%)	4 (18.2%)	
	Others/polymicrobial	34 (22.7%)	29 (22.7%)	5 (22.7%)	
Aerobes (mono or polymicrobial) ^a^	*S*. *aureus*	56 (37.3%)	47 (36.7%)	9 (40.9%)	0.7
	No *S aureus*	94 (62.7%)	81 (63.3%)	13 (59.1%)	
				
	Streptococci	24 (16.0%)	21 (16.4%)	3 (13.6%)	0.9
	No streptococci	126 (84.0%)	107 (83.6%)	19 (86.4%)	
				
	Gram-negative bacilli	43 (28.7%)	34 (26.6%)	9 (40.9%)	0.17
	No Gram-neg bacilli	107 (71.3%)	94 (73.4%)	13 (59.1%)	
Anaerobes ^a^	Yes	8 (5.3%)	7 (5.5%)	1 (4.5%)	1
	No	142 (94.7%)	121 (94.5%)	21 (95.5%)	
**Blood culture**					
Blood culture available	Yes	252 (41.6%)	217 (41.7%)	35 (40.7%)	0.9
	No	354 (58.4%)	303 (58.3%)	51 (59.3%)	
Results of culture	Positive	46 (18.3%)	40 (18.4%)	6 (17.1%)	0.9
	Negative	206 (81.7%)	177 (81.6%)	29 (82.9%)	
Positive culture	Monomicrobial	46 (100%)	40 (100%)	6 (100%)	-
	Polymicrobial	0 (0.0%)	0 (0.0%)	0 (0.0%)	
Aerobes ^b^	None	206 (81.7%)	177 (81.6%)	29 (82.9%)	0.7
	*S*. *aureus*	8 (3.2%)	6 (2.8%)	2 (5.7%)	
	Streptococci	18 (7.1%)	16 (7.4%)	2 (5.7%)	
	Gram-neg bacilli	7 (2.8%)	7 (3.2%)	0 (0.0%)	
	Others	13 (5.2%)	11 (5.1%)	2 (5.7%)	
Anaerobes	Yes	1 (0.4%)	1 (0.5%)	0 (0.0%)	0.7
	No	251 (99.6%)	216 (99.5%)	35 (100%)	
**Pus or blood culture**					
Any culture	Yes	333 (55.0%)	285 (54.8%)	48 (55.8%)	0.9
	No	273 (45.0%)	235 (45.2%)	38 (44.2%)	
Any microorganism identified	Yes	155 (25.6%)	133 (25.6%)	22 (25.6%)	1
	No	451 (74.4%)	387 (74.4%)	64 (74.4%)	
Specific microorganisms ^c^	None	178 (53.5%)	152 (53.3%)	26 (54.2%)	1
	*S*. *aureus* only	50 (15.0%)	43 (15.1%)	7 (14.6%)	
	Streptococci only	33 (9.9%)	28 (9.8%)	5 (10.4%)	
	Gram-neg bacilli only	28 (8.4%)	24 (8.4%)	4 (8.3%)	
	Others/polymicrobial	44 (13.2%)	38 (13.3%)	6 (12.5%)	
Aerobes (mono or polymicrobial) ^c^	*S*. *aureus*	61 (18.3%)	50 (17.5%)	11 (22.9%)	0.4
	No *S*. *aureus*	272 (81.7%)	235 (82.5%)	37 (77.1%)	
				
	Streptococci	40 (12.0%)	35 (12.3%)	5 (10.4%)	0.7
	No streptococci	293 (88.0%)	250 (87.7%)	43 (89.6%)	
				
	Gram-negative bacilli	50 (15.0%)	41 (14.4%)	9 (18.8%)	0.4
	No Gram-neg bacilli	283 (85.0%)	244 (85.6%)	39 (81.3%)	
Causing microorganisms identified	Yes	143 (23.6%)	122 (23.5%)	21 (24.4%)	0.9
	No	453 (74.8%)	389 (74.8%)	64 (74.4%)	
	Doubtful	10 (1.7%)	9 (1.7%)	1 (1.2%)	

^a^ Respect to all patients with pus

^b^ Respect to all patients with blood

^c^ Respect to all patients with any culture

Pus culture was available for 24.8% of the patients, and yielded positive results in 78.8% of them. A single microorganism was identified in most cases (78.0% of the positive cultures), and the most commonly recovered pathogen was *S*.*aureus*, either alone or in combination with others (30.0% and 37.3%, respectively, of the patients with culture). Anaerobes were uncommon (5.3%).

Blood culture was available for 41.6% of the patients, and was positive in 18.3%, in all cases for a single agent. Streptococci were the most commonly identified bacteria (7.1% of all blood cultures). Overall the causative microorganism was identified in 23.6% of all patients.

Table 4 depicts the cellulitis treatment.

**Table 4:** Cellulitis treatment.

		All (n = 606)	Good response (n = 520)	Poor response (n = 86)	P value
Treatment before admission	Yes	237 (39.1%)	211 (40.6%)	26 (30.2%)	0.07
	No	369 (60.9%)	309 (59.4%)	60 (69.8%)	
Initial treatment at admission ^a^	Single antibiotic	381 (62.9%)	322 (61.9%)	59 (68.6%)	0.2
	>1 antibiotic	225 (37.1%)	198 (38.1%)	27 (31.4%)	
Amoxicillin-clavulanate monotherapy	Yes	259 (42.7%)	220 (42.3%)	39 (45.3%)	0.6
	No	347 (57.3%)	300 (57.7%)	47 (54.7%)	
Change of the initial regimen	Yes	184 (30.4%)	159 (30.6%)	25 (29.1%)	0.8
	No	422 (69.6%)	361 (69.4%)	61 (70.9%)	
Reason for change ^b^	Culture	57 (31.0%)	48 (30.2%)	9 (36.0%)	0.8
	Poor response	55 (29.9%)	47 (29.6%)	8 (32.0%)	
	Toxicity	11 (6.0%)	9 (5.7%)	2 (8.0%)	
	Others	61 (33.2%)	55 (34.6%)	6 (24.0%)	
Days until change ^b^		3.5 (3.2–3.9)	3.5 (3.1–3.9)	3.5 (2.7–4.7)	0.9
Treatment after change ^b^	Single antibiotic	86 (46.7%)	77 (48.4%)	9 (36.0%)	0.2
	>1 antibiotic	98 (53.3%)	82 (51.6%)	16 (64.0%)	
Antibiotic treatment after discharge	Yes	504 (85.1%)	440 (84.6%)	64 (88.9%)	0.3
	No	88 (14.9%)	80 (15.4%)	8 (11.1%)	
Treatment after discharge	Single antibiotic	407 (80.8%)	356 (80.9%)	51 (79.7%)	0.8
	>1 antibiotic	97 (19.2%)	84 (19.1%)	13 (20.3%)	
Total number of antibiotics used		1.6 (1.5–1.7)	1.6 (1.5–1.7)	1.5 (1.4–1.67)	0.3
Total number of antibiotics used	1	289 (47.7%)	243 (46.7%)	46 (53.5%)	0.8
	2	191 (31.5%)	168 (32.3%)	23 (26.7%)	
	3	89 (14.7%)	77 (14.8%)	12 (14.0%)	
	4	35 (5.8%)	30 (5.8%)	5 (5.8%)	
	5	2 (0.3%)	2 (0.4%)	0 (0.0%)	
Days of IV antibiotic treatment		6.1 (5.8–6.5)	6.1 (5.7–6.5)	6.6 (5.6–7.9)	0.3
Total days of antibiotic treatment		13.3 (12.7–13.9)	13.3 (12.7–13.9)	13.1 (11.3–15.3)	0.9
Surgical treatment	Yes	81 (13.4%)	71 (13.7%)	10 (11.6%)	0.6
	No	525 (86.6%)	449 (86.3%)	76 (88.4%)	

Values are expressed as mean (95% CI) or % as appropriate.

^a^The most common monotherapy regimens were amoxicillin-clavulanate (259 patients, 42.7%), piperacillin-tazobactam (24 patients, 4.0%) and cefazolin (22 patients, 3.6%), whereas the most common combination therapy was clindamycin plus either ciprofloxacin or levofloxacin (40 patients, 6.6%).

^b^Only in patients who underwent treatment modification respect to the initial regimen

IV denotes intravenous

A substantial part of the patients (39.1%) were receiving antibiotics at the time of admission. The initial regimen at admission was based on a single antibiotic in most cases (62.9%), and amoxicillin-clavulanate monotherapy was the most commonly used regimen (42.7% of all patients). However, 30.4% of the patients required modification of the initial regimen, a mean of 3.49 days after the onset, due mainly to culture results (31.0%) or poor response (29.9%).

The rate of antibiotic monotherapy remained roughly similar in the 184 patients who underwent treatment modifications (50.5% vs 46.7%, respectively, P = 0.5). However, treatment was simplified to some extent in the 57 patients in whom the change was due to culture results (monotherapy 45.6% before vs 57.9% after change, P = 0.3), whereas treatment was intensified in the 55 patients who underwent change because of suboptimal responses (monotherapy 65.5% before vs 20.0% after treatment modification, P<0.0001).

Antibiotic therapy was continued after discharge in 85.1% of patients, most of them with a single antibiotic (80.8%). Overall, the patients received a mean of 1.59 different antibiotics for the treatment of cellulitis, during a mean of 13.3 days, approximately half of them by the IV route (6.14 days). Surgical interventions were relatively uncommon (13.4%).

#### Patients with good vs poor responses to treatment

The comparative, univariate analyses of the studied parameters according to the response are also reported in Tables 1 to 4. In summary, patients with good as compared with poor responses were younger (mean 62.7 vs 68 years, P = 0.03), had lower rates of prior cellulitis episodes (22.1% vs 47.7%, P<0.0001), and lower number of episodes in those with prior cellulitis (1.58 vs 2.19, P = 0.003).

Likewise, patients with good responses had more commonly non-surgical trauma and less commonly skin ulcers as predisposing factors (34.9% vs 15.4% and 4.7% vs 20.0%, respectively, P<0.0001), as well as prior skin lesions (28.1% vs 41.9%, P = 0.01), venous insufficiency (18.1% vs 34.9%, P = 0.0003), edema/lymphedema (24.4%vs 47.7%, P<0.0001), immunosuppression (10.0% vs 20.9%, P = 0.003) and diverse other comorbidities (72.5% vs 87.2%, P = 0.004).

Regarding the cellulitis episode (Table 2), patients with good vs poor responses had less involvement of lower extremities (72.3% vs 89.5%, P = 0.006), lower rates of sepsis (9.8% vs 16.3%, P = 0.07), and lower serum creatinine levels (1.02 vs 1.11 mg/dl, P = 0.09), hospital stays (6.86 vs 8.07 days, P = 0.052) and mortality (0.4% vs 18.6%, P<0.0001). On the contrary, there were no significant differences between the two groups from a microbiological (Table 3) and therapeutic (Table 4) perspective.

A logistic regression model was constructed using the variables with a P value <0.1 in the univariate analysis, excluding the vital outcome, to identify the factors independently associated with the response to therapy (Table 5).

**Table 5:** Variables independently associated with poor cellulitis outcome.

	OR (95% CI)	P value
Prior episodes of cellulitis ^a^	-	0.0001
1	1.6 (0.8–3.1)	0.2
2	1.4 (0.4–4.2)	0.6
3	6.6 (2.6–16.4)	0.0001
>3	4.3 (1.7–10.8)	0.002
Type of wound ^b^	-	0.06
Surgical	2.2 (0.9–5.6)	0.08
Non-surgical trauma	0.3 (0.1–0.8)	0.015
Skin ulcer	1.6 (0.9–2.9)	0.1
Venous insufficiency	2.3 (1.3–4.1)	0.004
Immunosuppression	2.1 (1.1–4.2)	0.03
Sepsis	2.0 (1.0–4.2)	0.05

^a^vs. no prior cellulitis

^b^vs no wound.

According to this model, which adequately fitted the data according to the Hosmer-Lemeshow goodness-of-fit statistic, the variables significantly predictive of poor responses were: three or more episodes of prior cellulitis, lower rates of non-surgical trauma, and presence of venous insufficiency, immunosuppression and sepsis.

The inclusion of vital outcome in the model yielded almost identical results, with the exception of sepsis, which was excluded (P = 0.6) because death was strongly associated with cellulitis outcome, although with wide 95% CI because of the small number of deaths (OR 142.9, 95% CI 17.9–714.3, P<0.0001).

## Discussion

In our large series of 606 adult patients, we found poor responses to treatment in 14.2% of the patients, a proportion similar to that found in some studies [5, 7], and lower than in others [18, 19]. We also found that prior episodes of cellulitis, venous insufficiency, immunosuppression and sepsis were independently associated with poorer outcomes of cellulitis, whereas recent non-surgical trauma was predictive of better outcomes, after adjusting for covariates. Interestingly neither the type of microorganism nor the number of antimicrobials administered or its duration associated with different response to therapy.

Although the history of prior episodes of cellulitis itself appears to be deleterious for the outcome of the infection, the number of such episodes seems to be critical. Thus, patients with one or two prior episodes had similar responses to treatment, whereas the outcome of patients with three or more episodes was clearly worse. Taking into account that 95.1% of recurrent cellulitis in the poor response group occurred in the same location, it can be derived that successive episodes of cellulitis could lead to residual lymphatic and/ or microvascular damage, probably accentuated by sustained inflammation or fibrosis. Consequently, the treatment of further episodes would be less successful. Our findings of poorer outcomes associated with venous insufficiency would also support this explanation.

A history of one or more episodes of cellulitis was observed in 25.7% of our patients, a proportion concordant with the 18% to 49% reported in several studies [3–5, 20, 21]. Although the convenience of chronic administration of antibiotics to prevent recurrences is controversial [3–5], some studies, including a meta-analysis, found that regimens composed of daily oral penicillin or monthly intramuscular benzathine penicillin were efficacious to reduce the recurrence rate or the time to recurrence under certain circumstances [15, 22–24]. Therefore, this option should be considered in patients with multiple episodes of cellulitis.

In fact, the 2014 guidelines from the Infectious Diseases Society of America indicate that treatment with oral penicillin or erythromycin or intramuscular benzathine penicillin should be considered in patients who have 3–4 episodes of cellulitis per year, despite attempts to treat or control predisposing factors, although the strength for this recommendation was weak and the quality of evidence only moderate [8]. However, in some cases cellulitis might recur, even with antibiotics prophylaxis, and the protective effect diminished progressively once the prophylaxis was stopped [22, 24].

Our results suggest an additional perspective for the convenience of preventive treatment in patients with highly recurrent cellulitis, as the response to treatment seems to be somewhat impaired in these cases, and poor cellulitis responses were strongly related to poor vital outcomes in the univariate and multivariate analyses. From this perspective, the best benefits would be expected if antimicrobial prophylaxis prevented the development of more than two recurrent episodes in the same location.

Venous insufficiency leading to lower limbs edema, chronic lymphedema and chronic obstructive venous disease are well-known vascular factors favoring cellulitis [3, 5, 14, 16]. The presence of edema/lymphedema and venous insufficiency were significantly associated with poor responses to cellulitis therapy in our study. However, only the latter was independently predictive of such outcome, suggesting that the vascular component, rather than the edema itself, was responsible for the worse response to treatment.

Not surprisingly, the other predisposing factor independently associated with poor cellulitis outcome in the multivariate analysis was immunosuppression. On the contrary, non-surgical trauma was the only factor significantly predictive of better cellulitis outcomes, a finding that could be explained by the lower presence of underlying conditions, predisposing factors (especially edema/lymphedema), and comorbidities observed in the patients with this particular type of cellulitis.

A retrospective study on 106 evaluable cases found that inappropriate antimicrobial selection and dosing, prior antibiotic treatment and marked obesity were associated with poorer outcomes [19]. Another retrospective study used ICD-9 codes to identify 293 outpatients with uncomplicated cellulitis and found obesity and heart failure to be associated with treatment failure [18]. Our prospective study that evaluated a large number of variables of 606 hospitalized patients failed to confirm such associations. However, the comparison among studies may be difficult because of different designs, settings, statistical power, covariates analyzed and criteria for failure. In fact, the failure rates in these studies (32.1% and 24%, respectively) were appreciably higher than in ours (14.2%) and the degree of obesity associated with failure in the two studies, as evaluated by the body mass index, was considerably higher than in our series.

Regarding microbiological issues, the causing microorganisms are rarely identified in cellulitis, with rates of about 15–30% [4, 5]. We found positive pus cultures in 19.5% of the patients as a whole (78.7% of the 150 patients with available culture). Most of the positive cultures were monomicrobial (78.0%), and *S*. *aureus* was the most commonly isolated pathogen (37.3% of all cultures). Although streptococci are usually considered to be somewhat more common than *S*. *aureus* [4, 8], the etiology may vary depending on diverse circumstances [3–6], and *S*. *aureus* is recovered with higher frequency from purulent collections [4, 9]. Our results support this statement and, in fact, we found streptococci to be somewhat more common than *S*. *aureus* in blood cultures.

The yield of blood cultures is considerably lower, with values of about 2–8% [4–6, 10, 25, 26] and, therefore, they are not routinely recommended in recent guidelines, except for certain specific groups [5, 8]. We found positive results in 18.3% of patients with blood culture available (7.6% of the patients as a whole), all of them monomicrobial. Like ours, other studies found streptococci to be more common than *S*. *aureus* in blood cultures [10, 26].

Overall, the causing microorganism was identified, either in pus or blood, in about one-fourth of the patients in our study, without any difference regarding the type of response to treatment. Community-acquired MRSA infection rate is increasing. A study from Hawaii, one of the world places with higher MRSA prevalence, reported 62% of MRSA isolates in patients with abscesses or skin ulcers [27]. In two studies of skin and soft tissue infections in adults from Spain, *S*. *aureus* was isolated in 35.1% and MRSA in 12.9% of patients [13], whereas in other smaller study MRSA reached 22% [28]. In our multicenter series, MRSA represented 24.6% of all pure or mixed isolates of *S*. *aureus* in blood or pus (9.8% of all patients with positive cultures), without any significant difference in the response to treatment as compared with methicillin-sensitive *S*. *aureus* (P = 0.8).

Of note, none of the multiple microbiological items studied had any significant association with the response to treatment. Similarly, none of the diverse parameters analyzed related to treatment, including the number, duration or administration route of the antibiotics, as well as the surgical treatment, had any significant impact on the cellulitis outcome in the univariate or multivariate analyses.

Likewise, none of the clinical, laboratory, imaging or hospitalization parameters were associated with the response to therapy in the multivariate analysis, with the exception of sepsis, which was marginally associated with this outcome (P = 0.05), but was excluded (P = 0.6) when a stronger and related variable, the vital outcome, was entered into the model (P<0.0001).

The main strengths of our study are the large number of patients included, its prospective nature and the large number of parameters evaluated, which allowed to adjust for and minimize the effect of confounding variables. Limitations include the lack of a unified treatment protocol, to evaluate the responses to the same drugs, and the variability inherent to multicenter studies, particularly regarding procedural and management issues. However, no unified treatment protocol exists currently [3–6, 8], given the diversity of clinical and microbiological circumstances inherent to cellulitis, and the multicenter nature of the study allows to evaluate the real clinical practice across our country, minimizing potential biases from a single institution.Finally, given the setting of our study, these results may only be generalizable to hospitalized patients and not to a community based or outpatient population.

From our large study, we conclude that three or more episodes of prior cellulitis, venous insufficiency, immunosuppression, and the development of sepsis are independently associated with poorer cellulitis outcomes, whereas microbiological, clinical and therapeutic aspects were not. Beyond the possible role of antibiotic prophylaxis for the prevention of recurrences, its use could also be useful to improve the response to treatment in patients with previous multiple episodes of cellulitis that develop new events.
